# Is It Possible to Replace Conventional Radiography (CR) with a Dose Neutral Computed Tomography (CT) of the Cervical Spine in Emergency Radiology—An Experimental Cadaver Study

**DOI:** 10.3390/diagnostics12081872

**Published:** 2022-08-02

**Authors:** Zsuzsanna Deak, Lindis Brummund, Sonja Kirchhoff, Markus Körner, Lucas Geyer, Fabian Mück, Mariano Scaglione, Maximilian Reiser, Ulrich Linsenmaier

**Affiliations:** 1 Imaging Urania, Laurenzenberg 2, 1010 Wien, Austria; dr.deak.zsuzsanna@gmail.com; 2 Departmant of Radiology, University Hospital LMU, Marchioninistraße 15, 81377 Munich, Germany; lindis.brummund@gmx.de (L.B.); markus.koerner@muehleninsel.de (M.K.); geyer@rzm.de (L.G.); fabian.mueck@helios-gesundheit.de (F.M.); maximilian.reiser@med.uni-muenchen.de (M.R.); 3 Department of Forensic Medicine, Ludwig-Maximilians-University of Munich, Nußbaumstraße 26, 80336 Munich, Germany; sonja.kirchhoff@kernspin-maximilianstrasse.de; 4 Radiologische Praxis Maximilianstraße, Maximilianstr. 34, 84028 Munich, Germany; 5 Radiologie Mühleninsel, Mühlenstraße 4, 84028 Landshut, Germany; 6 Radiologisches Zentrum München, Pippinger Straße 25, 81245 Munich, Germany; 7 Institute for Diagnostic and Interventional Radiology, HELIOS Kliniken München West, München Perlach & Augustinum München, Steinerweg 5, 81241 Munich, Germany; 8 Department of Radiology, University of Sassary, 07100 Sassari, Italy; mscaglione@uniss.it; 9 James Cook University Hospital, Marton Road, Middlesbrough TS4 3BW, UK

**Keywords:** cervical spine, trauma patients, computed tomography, dose index, computed tomography, conventional radiography, emergency radiology

## Abstract

The purpose of this experimental study on recently deceased human cadavers was to investigate whether (I) the radiation exposure of the cervical spine CT can be reduced comparable to a dose level of conventional radiography (CR); and (II) whether and which human body parameters can be predictive for higher dose reduction potential (in this context). Materials and Methods: Seventy serial CT scans of the cervical spine of 10 human cadavers undergoing postmortem virtual autopsy were taken using stepwise decreasing upper limits of the tube current (300 mAs, 150 mAs, 110 mAs, 80 mAs, 60 mAs, 40 mAs, and 20 mAs) at 120 kVp. An additional scan acquired at a fixed tube current of 300 mAs served as a reference. Images were reconstructed with filtered back projection and the upper (C1-4) and lower (C4-7) cervical spine were evaluated by three blinded readers for image quality, regarding diagnostic value and resolution of anatomical structures according to a semiquantitative three-point-scale. Dose values and individual physical parameters were recorded. The relationship of diagnostic IQ, dose reduction level, and patients’ physical parameters were investigated. The high-contrast resolution of the applied CT protocols was tested in an additional phantom study. Results: The IQ of the upper cervical spine was diagnostic at 1.69 ± 0.58 mGy (CTDI) corresponding to 0.20 ± 0.07 mSv (effective dose) in all cadavers. IQ of the lower cervical spine was diagnostic at 4.77 ± 1.86 mGy corresponding to 0.560 ± 0.21 mSv (effective dose) in seven cadavers and at 2.60 ± 0.93 mGy corresponding to 0.31 ± 0.11 mSv in four cadavers. Significant correlation was detected for BMI (0.8366; *p* = 0.002548) and the anteroposterior (a.p.) chest diameter (0.8363; *p* = 0.002566), shoulder positioning (0.79799; *p* = 0.00995), and radiation exposure. Conclusions: Conventional radiography can be replaced with a nearly dose-neutral CT scan of the cervical spine.

## 1. Introduction

In emergency radiology, the number of trauma patients at risk for injury to the cervical spine (c-spine) is high, but the percentage of significant injuries is relatively low, resulting in a large number of normal studies undertaken in clinical practices around the world. The reported incidence of c-spine injuries in association with brain injuries among adult trauma patients ranges from 1.7 to 8%, and it is even lower than 1% in alert, neurologically intact patients; however, missed injuries can have serious consequences [1,2,3]. Established clinical indication rules for imaging, such as CCR (Canadian Cervical Spine Rule) or NEXUS (National Emergency X-Radiography Utilization Study), intend to lower the number of unnecessary normal imaging tests by keeping levels of specificity and sensitivity at reasonable and effective clinical levels [1,2,3,4,5,6,7]. According to CCR and NEXUS, conventional radiography (CR) is regularly to be applied only in low-risk patients, and all other patients, considered as high risk, should undergo computed tomography (CT) imaging [6,7], which is nowadays an accepted clinical practice. The American College of Radiology (ACR) therefore also favors in their appropriateness criteria CT as the imaging modality of choice in patients at risk according to NEXUS or CCR [8]. However, Bailitz et al. reported in a prospective study of 1583 patients a very low sensitivity of 25% for CR in comparison to CT even in low-risk patients for detecting spinal injuries [9].

Multiple studies have established that CT has a dramatically improved diagnostic sensitivity (98%) in comparison to the variable sensitivity (52%) of conventional radiography in the trauma patient [10,11,12]. Since there has been a shift of paradigms away from the use of CR, the demand of CT in patients with suspected c-spine trauma has very much been on the rise in clinical routine [13,14]. For example, Broder et al. reported an increase of 463% of c-spine CT over a period from 2000 to 2006, corresponding to an exponential growth rate with an annual increase of up to 15% [15]. The growing importance of CT, now an easily and widely available diagnostic technique for trauma patients, has resulted in an increase of exposure to radiation besides better diagnosis and improved patient care. Current developments have been still focusing on dose reduction with promising experiences in the CT imaging of the c-spine [16,17,18,19,20]. Initial results indicate that sub-millisievert (<1.0 mSv) CT scans of the cervical spine after trauma are possible [18]. 

The purpose of our experimental study on recently deceased human cadavers was to investigate whether the radiation exposure of c-spine CT can be reduced to the dose level of CR, and if certain physical body parameters [PBP) are predictive for higher dose reduction potentials.

## 2. Materials and Methods 

### 2.1. Study Design

In our experimental postmortem cadaver study, consecutive dose reduction was applied and image quality (IQ) was evaluated to assess the individually sufficient radiation dose for diagnostic CT studies. This “individualized” or personalized medicine study concept included the differentiation and characterization of physical body parameters (PBP) of the examined cadavers to correlate with the lowest diagnostically still-applicable individual CT dose values. The acquisition parameters of the dose-reduced protocols were also evaluated in a phantom study to quantify the decrease of objectively obtained high-contrast resolution, comparable to the CT imaging of bony structures in the c-spine in clinical studies. The post mortem CT (PMCT) examinations were performed in deceased trauma patients after being transferred for virtual autopsy upon the written request of the general prosecutor in a cooperation with the academic institute for forensic medicine. The study was approved by the local ethics committee and a waiver of consent was provided by the institutional medical advisory board as well as by the general prosecutor’s office.

### 2.2. Human Cadaver Study

Ten cadavers of consecutive deceased patients (age: 41.4 ± 9.5 years, range: 28–53 years; gender: male:female = 4:1) transferred for postmortem virtual autopsy underwent the following CT protocols. All imaging studies were performed on a 64-detector-row CT scanner (Discovery HD750; GE Healthcare, Waukesha, WI, USA). Six (level 1–6) consecutively dose-reduced experimental scans followed the standard scan using a noise index of 42 and an upper threshold of the tube current of 300 mA. The standard protocol is identical to our clinical routine PMCT protocol. In the six experimental protocols, the upper threshold of the tube current was therefore stepwise lowered to 150 mAs, 110 mAs, 80 mAs, 60 mAs, 40 mAs, and 20 mAs to evaluate the lowest possible dose reduction level for possible clinical study trials. The reference scan was performed using fixed mAs without automatic tube current modulation; it served as a reference and represented the optimal image quality c-spine CT study. All other parameters like tube voltage (120 kVp), rotation time (0.8 s), pitch (0.531:1), and detector collimation (20 mm) were kept at constant levels. The c-spine was scanned from the first thoracic (T1) vertebral body to the contour of the foramen magnum (C0). Images were reconstructed exclusively with filtered back projection in bone kernels as thin slices of 0.625 mm using a field of view of 24 cm and a matrix of 512 × 512 pixels; axial and sagittal images of 2 mm were reformatted as multiplanar reconstruction (MPR).

### 2.3. Evaluation of IQ and Physical Body Parameters

The anonymized CT images were transferred to the institutional picture archiving and communication system (PACS). Subjective IQ was evaluated independently by three radiologists (with 2, 8, and 15 years of experience; initials: L.G., M.K., U.L.).

The upper and lower region of the c-spine were separately analyzed, because the depiction of the injuries to the lower c-spine (C4–C7 vertebras) is often impaired by artifacts such as beam hardening and photon starvation artifacts due to the individual shoulder girdle anatomy. For evaluation in the upper region, the C3, in the lower cervical region, the C7 vertebra were exemplary selected. Both vertebrae were separately evaluated for spongious and cortical bone structure with regard to sharpness of the bony architecture both in axial and sagittal planes [21,22,23]. IQ was evaluated with a semi-quantitative rating scale (0: non-diagnostic; 1: diagnostic/sufficient; 2: diagnostic/excellent) with regard to efficiency of ruling out fractures in comparable clinical settings. At different dose levels for all patients, the median values of IQ ratings, including the scoring of both cortical and spongious bone, were calculated, again, separately, for axial and sagittal reformations, and for C3 and the critical C7 vertebra levels. If any of the three observers assessed an image as non-diagnostic, the overall IQ was rated as non-diagnostic. With those results, the ratings of every individual scan protocol still providing diagnostic IQ at the lowest possible radiation dose was identified for each patient.

Shoulder distance (lateral diameter (cm) and anteroposterior chest diameter (anteroposterior diameter (cm)) were measured at the level of the jugular fossa, and body mass index (BMI) was calculated from weight (kg) and height (cm), in order to estimate the influence of different physical body characteristics on IQ, their individual expressions in this study cohort, and influences of the latter on the experimental protocols. The position of the shoulder girdle was defined by assessing the level of the shoulder outline projecting to the c-spine using the lateral scout view. A nominal value was given with reference to the base plate of the seventh vertebra as “0” and the upper contour of the atlas as “7”. For example, if the shoulder outline projected to the disc between C3 and C4, the nominal value was 4; if projected to the C4 vertebra, the nominal value was “3.5”. One of the cadavers presented with a declined shoulder position—so called “swimmer’s” position [22,23,24,25]. This cadaver was excluded for the statistical analysis of the effect of shoulder position on the reduction of radiation exposure.

### 2.4. Phantom Study

In a phantom study, high-contrast resolutions of the different study protocols were compared with the CTP528 module of a CATPHAN600^®^ (The Phantom Laboratory, Greenwich, NY, USA). The scanning of the phantom at a fixed tube current of 300 mAs, again serving as a reference, was followed by a series (n = 7) of dose-reduced scans from 150 mAs, 110 mAs, 80 mAs, 60 mAs, 40 mAs, 20 mAs, and 1 0mAs. All other parameters were kept constant and identical to the cadaver study. Images were reconstructed exclusively with filtered back projection in a bony kernel with a slice thickness of 0.625 mm and reformatted with a slice thickness (ST) of ST = 2 mm, comparable and identical to the cadaver study. High-contrast resolution was assessed by counting the identifiable line pairs of the phantom test gauge per cm [LP/cm]. Five independent readers blinded to scan parameters separately evaluated the image resolution. As a result of the five ratings, the lowest resolution for each protocol was accepted.

### 2.5. Estimation of Dose Values

Dose reports including scan parameters of CT dose index (CTDI) were recorded for each scan in phantom and cadaver studies and dose length product (DLP) in the cadaver studies as well. The assessment of the size-specific dose estimate is not available for c-spine, so the effective dose (ED) was estimated by multiplying DLP with the corresponding conversion factor (E_DLP_ = 0.0051 mSv/mGy/cm) [26]. 

### 2.6. Statistical Analysis

CTDI, DLP, and ED were descriptively analyzed with mean value, standard deviations, and minimum and maximum values. The image quality (IQ) ratings of the standard protocol and dose-reduced protocols were compared with the reference protocol using a Wilcoxon signed-rank test. The level of significance was set at 0.05 (*p* value) and adjusted according to Bonferroni correction (0.05/4 = 0.0125) for four comparisons (axial and sagittal images of C3 and C7 vertebra). The consistency of the subjective ratings of the radiologists was estimated by calculating Cronbach’s Alpha correlation coefficient. The linear relationship between the diagnostic scan protocol of the lowest dose level (CTDI values) and patients’ different physical characteristics (BMI, lateral and anteroposterior diameter, and shoulder position) was investigated by calculating the Pearson product-moment or Spearman’s rank correlation coefficient depending on data distribution. The *p* value (0.05) for the characterization of four body parameters was adjusted according to Bonferroni correction (0.05/4 = 0.0125). Statistical Package für Social Sciences (SPSS18.0.0) was used for all calculations (PASW, IBM, Armonk, NY, USA).

## 3. Results 

### 3.1. Cadaver Study

The detailed dose values of all protocols including the phantom and cadaver studies are listed in Table 1. The lowest effective dose levels were 0.20 mSv (SD ± 0.07) at 10 mA tube current; the highest were at 4.11 mSv (SD ± 1.29) at 300 mA, and therefore 20.6 fold higher, representing the reasonable experimental dose spectrum for investigating c-spine CT dose reduction and potentials in clinical radiology as well. The mean values of the CTDI (mGy), DLP (mGyx m), and ED (mSv) were 12.44 ± 4.56, 287 ± 107, and 1.46 ± 0.55, respectively, in the standard protocol using tube current modulation with an upper limit of 300 mAs.

**Figure 1:**
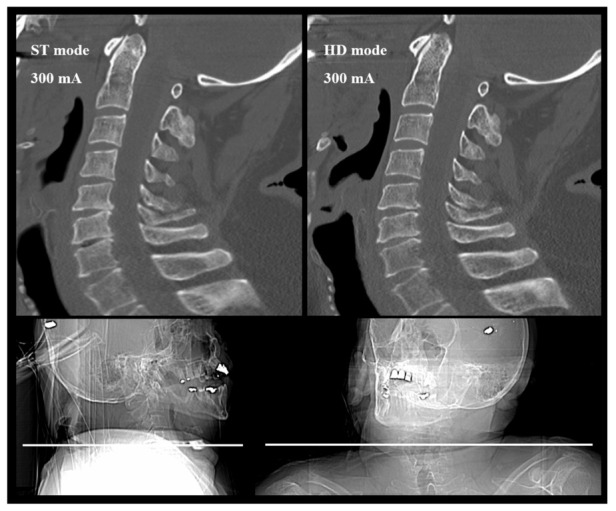
Image quality of the upper c-spine (C1–C4) is diagnostic regardless of all body parameters with protocol VI at 20 mAs. In this cadaver (BMI of 30.9 kg/m^2^, an anteroposterior diameter of 26 cm and high shoulder position), diagnostic images of the lower c-spine were acquired at 300 mAs with tube current modulation corresponding to a CTDI of 15.93 mGy and an effective dose of 1.59 mSv. Associated topograms of the same cadaver are shown in Figure 2.

**Figure 2:**
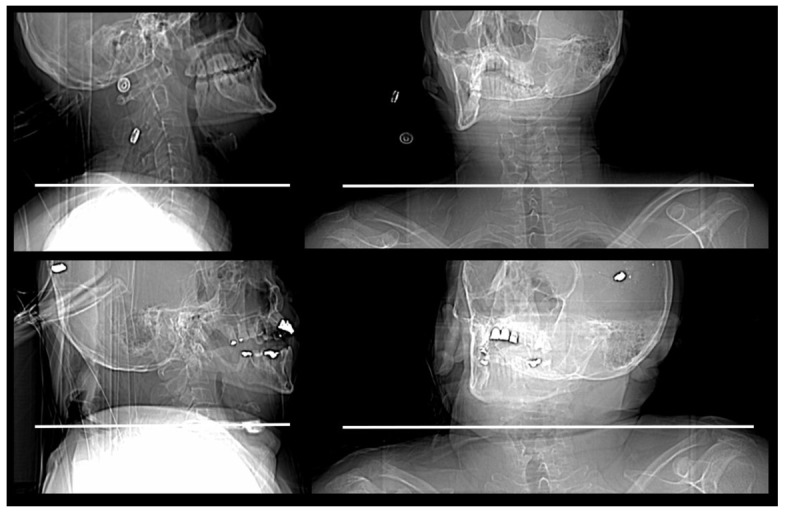
Lateral and anteroposterior topograms are presented and shoulder position are indicated in the same cadavers presented in Figure 1 and Figure 3. The upper topograms belong to patients of Figure 1 and the lower ones to the patient of Figure 3.

Data of IQ evaluation and *p* values are shown in Table 2. In comparison to the reference scan without tube current modulation, the standard protocol allowed for a comparable IQ for both the lower and upper c-spine in both axial and sagittal planes; the corresponding *p* values were >0.99999, >0.99999, 0.15700, and 0.52700, respectively. The upper c-spine, represented by the evaluation of the C3 area, was depicted with a comparable IQ (*p* = 0.01600) at 0.31 ± 0.11 mSv (CTDI: 2.60 ± 0.93 mGy; DLP: 60 ± 21 mGy*cm) in the axial plane and with significantly lower but diagnostic IQ at 0.20±0.07 mSv (CTDI: 1.69 ± 0.58 mGy; DLP: 39 ± 13 mGy*cm) in the axial (*p* = 0.00200) and sagittal planes (*p* < 0.00001). IQs of the reference, the standard, and the protocol of the lowest dose level are compared in Figure 1, demonstrating an obese cadaver with high shoulder position. Its shoulder posture is shown in Figure 2.

**Figure 3:**
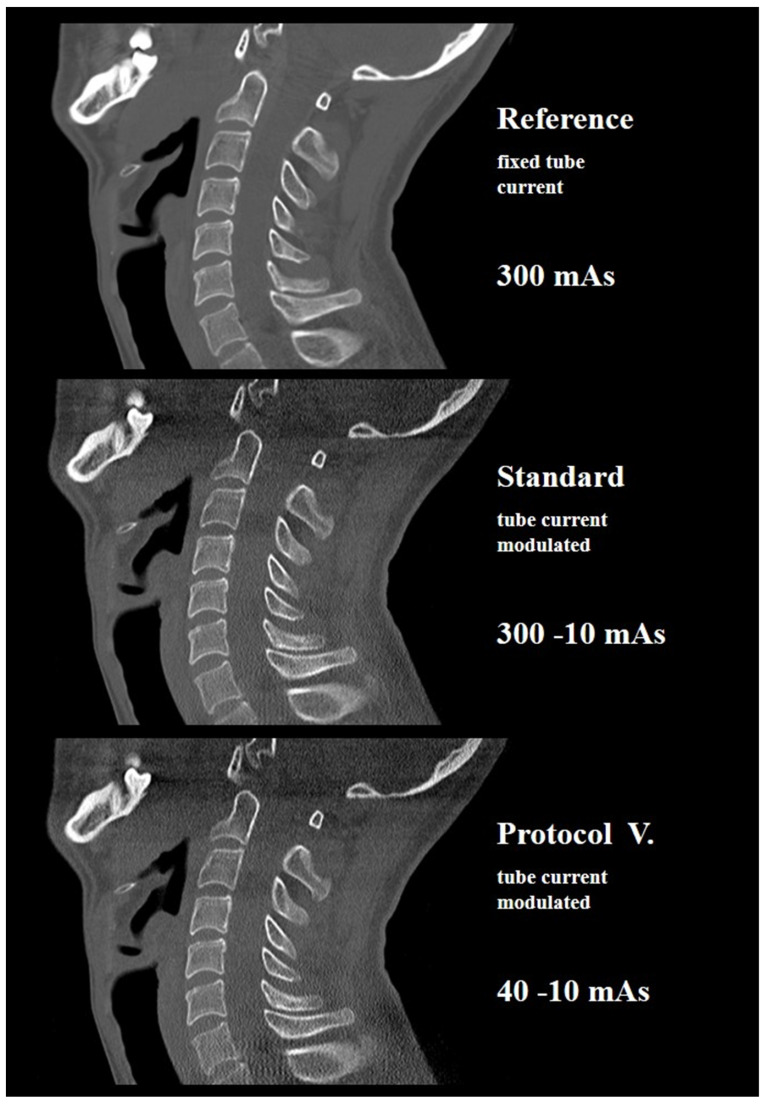
Diagnostic image quality was available with protocol V in this cadaver with a lowered shoulder and presenting with a BMI of 21.9 kg/m^2^ and an anteroposterior chest diameter of 17 cm, corresponding to a CTDI of 2.56 mGy and an effective dose of 0.30 mSv. Associated topograms of the same cadaver are shown in Figure 2.

The lower c-spine, represented by the C7 area, had limited potential for dose reduction. However, in 4 out of 10 human cadavers, the tube current could be decreased to 40 mAs, corresponding to a CTDI, DLP, and ED of 2.60 ± 0.93 mGy, 60 ± 21 mGy*cm, and 0.31 ± 0.11 mSv, respectively. (See also Figure 2 and Figure 3). Furthermore, in 7 out of 10 human cadavers, the lower c-spine could be depicted with diagnostic IQ at 110 mAs corresponding to a CTDI, DLP, and ED of 4.77 ± 1.86, 109 ± 42 mGy*cm and 0.56 ± 0.21 mSv, respectively. 

The values of Cronbach’s Alpha correlation coefficients ranged from 0.729 to 1.000, indicating an acceptable to excellent consistency of the IQ ratings; exact values are displayed in Table 2.

Detailed data of the ten cadavers and corresponding individual diagnostic protocol at the lowest possible radiation dose are displayed in Table 3. The calculated Pearson correlation coefficients for BMI, lateral, and anteroposterior diameter were 0.8366 (*p* = 0.002548), 0.6097 (*p* = 0.061276), and 0.8363 (*p* = 0.002566), respectively. The Spearman’s correlation coefficient was 0.79799 (*p* = 0.00995) for shoulder position. These data indicate that BMI, anteroposterior diameter, and shoulder position had a significant effect on radiation exposure and diagnostic IQ of the lower c-spine in our cadaver study. In two of ten cadavers presented with vertebral fractures, both classified as stable minor injuries of isolated endplates (type A.1) according to the AO classification scheme. In our still-limited number of cadavers, a preliminary cut-off value could be recognized at a BMI ≤ 25.5 kg/m^2^ regardless of other body parameters for the protocol N° II and at a BMI ≤ 21.9 kg/m^2^, an anteroposterior diameter ≤20 cm, and if the shoulder did not superimpose the C4 vertebra for the protocol N° V.

### 3.2. Phantom Study

**Figure 4:**
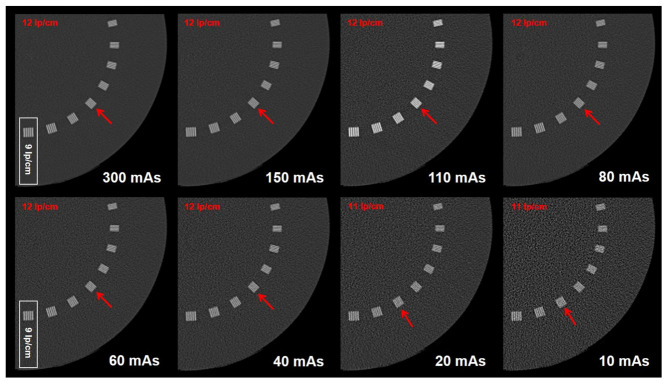
The images show the high-contrast resolution (red arrows) using a ***Catphan***^®^ Phantom (The ***Phantom*** Laboratory) for the phantom study at different tube current levels, thereby continuously decreasing the tube current in eight steps from 300 mA (reference standard) to 10 mA.

The CTDI was 34.14 mGy at 300 mAs and decreased to 1.16 mGy at 10 mAs. The dose values and tube current of the protocols are listed in Table 1. At 300 mA, the phantom study revealed a maximal high-contrast resolution of 12 LP/cm, corresponding to a spatial resolution of 0.42 mm. At 150, 110, 80, 60, and 40 mAs, the high-contrast resolution was still 12 LP/cm (0.42 mm), and at 20 and 10 mAs, it decreased to 11 LP/cm (0.45 mm), the results are detailed in Table 4 and highlighted in Figure 4. The phantom study indicates a high potential for dose reduction on the cost of relatively little impairment of the high-contrast resolution.

## 4. Discussion

Data published by Niiniviita et al. in 2018 show that the mean radiation dose of CT of the cervical spine in adult and pediatric patients of a Finnish emergency department was at an astonishing high 18.3 mGy over a 2-year period according to dose-monitoring software [27]. Data from reviewing doses to patients undergoing cervical spine CT examinations in the UK published in 2018 indicate a rounded average dose of 20 mGy for c-spine CT [28]. The mean radiation dose of our standard protocol—a protocol identical to the routine protocol in the clinical setting for c-spine trauma imaging at our department—was 12.44 ± 4.56 mGy. These dose values are lower than the recently published survey data and indicate the high potential and the urgent need of dose optimization in the CT imaging of the c-spine in trauma patients and applying and developing more individualized scan protocols.

The results of our dose-reduced protocols showed that diagnostic CT of the upper c-spine was ensured at a reduced tube current as low as 20 mAs at 1.69 ± 0.58 mGy, corresponding to 0.20 ± 0.07 mSv in adults independently of any physical body parameter. According to actual guidelines, the ED of c-spine radiography alternates from 0.01 to 0.1 mSv [29]. Mettler et al. reported 0.2 mSv, and results of a local survey in Greece, published recently, reported an even lower mean value of 0.06 mSv for two plain radiographs [30,31]. The use of a dedicated dose-optimized CT scan of the upper c-spine or a split protocol for upper and lower c-spine are under discussion to achieve best dose-reduction results. In these special cases, CT might completely replace conventional radiography of the c-spine at a dose-neutral level. Different scan parameters could be used, but images need to be fused in postprocessing and practicability is not proven.

The evaluation of the different body parameters of the human cadavers showed significant and strong correlation between BMI, anteroposterior chest diameter, shoulder position, and radiation exposure associated with the imaging of the lower c-spine. The differences among the correlation coefficients of these three parameters were low and they are probably not independent of one another. However, we see large potentials to integrate those parameters, which are easy to measure, in the steering software of future presets of individualized CT imaging protocols. For example, obese patients probably present with larger anteroposterior chest diameters and have more difficulties with pulling down their shoulder. However, trauma patients, if they are young or middle-aged adults, could easily cooperate to create more favorable imaging circumstances, especially considering that healthy adults usually have a shoulder posture that does not superimpose the upright cervical spine in a standing position [32,33]. Physical parameters like BMI, anteroposterior diameter, and shoulder position can be easily evaluated in the clinical setting by asking the patients and looking at the scout and should also be integrated in future CT scanner software. 

In patients with favorable physical parameters, like a pulled-down position of a shoulder (if C4 or even C5 were not superimposed by soft tissue on the lateral scout view) or lower normal BMI (<22 kg/m^2^) and lower anteroposterior shoulder diameter (≤20 cm), diagnostic CT imaging was possible at 2.60 ± 0.93 mGy, corresponding to 0.31 ± 0.11 mSv in our experimental study. To the best of our knowledge, the lowest possible radiation dose for the c-spine CT was 0.7 mSv, reported by Weinrich et al. in their feasibility study on human cadavers using 140 kVp [18]. Tozakidou et al. reported a dose-reduction potential down to 0.8 mSv in their dose finding study using four cadavers [20]. However, in 7 of 10 cadavers presenting with approximately normal BMI values (≤25.5 kg/m^2^), diagnostic IQ was available in our study regardless of shoulder position at a CTDI of 4.77 ± 1.86 mGy, corresponding to an ED of 0.56 ± 0.21 mSv, achieving still another 20 to 30% decrease to the latter-mentioned publications using tube current modulation with an upper limit of 110 mAs. These dose values are lower than any other reported before and suggest that more effective dose-optimized CT protocols of the c-spine could be established for normal patients of healthy weight and for patients with lower-than-normal BMI (<22 kg/m^2^) and lower anteroposterior shoulder diameter (≤20 cm) in emergency departments. However, at the same time, these results indicate the need to adapt protocols more individually in respect to individual anatomic settings. In our cadaver study, the optimization of shoulder position, like pulling down the shoulder, was not possible. In the sagittal scout views, the shoulder superimposed the C5, C6, and C7 vertebrae in 5 of 10 cadavers. An even more effective dose reduction could be expected in compliant trauma patients, if C5, C6, or even C7 are not superimposed by a shoulder girdle. For example, Tozakidou et al. have already reported a dose reduction of 51% for c-spine CT in trauma patients with shoulder pull-down [19]. In summary, imaging technique, patient positioning, and more advanced, preplanned anatomically (BMI, shoulder distance, etc.) individualized scan protocols have a further impact on dose reduction in c-spine imaging. However, in clinical trauma imaging, the influence of pulsations, breathing, and patient movements make a conceptual difference to the cadaver study results. Advances in preclinical cadaver imaging need further proof in the clinical routine setting. Additionally, CT scan speed, pitch, and rotation time of the scanner must be also adjusted, respectively, to minimize possible motion image artifacts.

Our phantom study served as a gold standard for the feasibility of the dose-reduced protocols, regarding the high-contrast resolution. The results of the phantom study confirm the results achieved in the cadaver study, indicating that the tube current could be decreased to 40 mAs without relevant impairment of the high-contrast resolution. The phantom study aims to present controlled and reproducible data on high-contrast resolutions of the applied protocols to allow for a possible adjustment of high-contrast resolutions of dose-reduced clinical protocols at different CT platforms. For the same reason, our study consequently excluded the use of any iterative reconstruction, but included those CT features like low collimation (20 mm) and low pitch, which improve high-contrast resolution and minimize artifacts [34]. The combination of a collimation of 20 mm and a pitch of 0.531 means that the 10-times-the-length of the collimation (200 mm) can be depicted within approximately 19 (18.868) rotations considering that a pitch of 0.531 means 10.6 mm table feed per rotation. Using a rotation time of 0.8 sec allows for a complete scan time of 15.1 sec (0.8 * 18.868). This means, using a tube current of 10 mAs or 40 mAs, the applied tube current is a maximal 151 mA or 604 mA over a scan length of 200 mm, corresponding to a total of 3.75 mA or 15 mA tube current for the length of the applied 10.6 mm table feed during one rotation in this study. By adapting the values of pitch, tube current, and rotation time, radiation exposure and high-contrast resolution could be adjusted to establish comparable protocols for other CT platforms.

The inclusion of only ten cadavers in this pilot study represents a limitation. However, this was to some extent coped with by an individualized and intra-individual study concept highlighting further fields and parameters of optimization of CT imaging of the c-spine. Another limitation was the relatively small number of c-spine fractures in our study population: two of ten cadavers presented with vertebral fractures, which were minor compressions of single endplates (AO A1 fracture type). Dose values of the scout scans were not available and not considered. Objective image noise was not measured, because noise measurements for air or soft tissue are not representative for high-contrast structures, like bony structures. We addressed this limitation in analyzing the high-contrast resolution in the phantom study. 

As a conclusion based on the results of our experimental study on postmortem human cadaver specimens, we propose that radiation exposure of diagnostic CT imaging of the complete c-spine could be decreased well under 1 mSv down to 0.56 mSv in those humans presenting with normal physical parameters like normal BMI. The combination of optimal shoulder positioning, lower BMI values, and low anteroposterior chest diameter allowed for a further decrease of radiation exposure down to 0.31 mSv. Our data suggest for the first time that a nearly dose-neutral replacement of the conventional radiography by CT is possible for the upper c-spine at a radiation exposure of 0.20 mSv regardless of variable body habitus.

## Figures and Tables

**Table 1:** Radiation doses of the phantom and patient studies; the mean values ± standard deviations and the ranges of CT dose index, dose length product, and effective dose are given for all scan protocols.

Phantom Study			Patient Study										
Protocol no.	Tube Current (mAs)	CT Dose Index Volume (mGy)	Protocol no.	Tube Current (mAs)	CT Dose Index Volume (mGy)			Dose Length Product (cm*mGy)			Effective Dose (mSv)		
					Mean SD	Range Min Max		Mean SD	Range Min Max		Mean SD	Range Min Max	
**I.**	300	34.82	**REFERENCE**	300	34.14 ± 0	34.14	34.14	733 ± 253	679	953	4.11 ± 1.29	3.47	4.87
**II.**	150	17.41	**STANDARD**	300–10	12.44 ± 4.56	10.25	16.19	287 ± 107	198	374	1.46 ± 0.55	1.01	1.91
**III.**	110	12.77	**I.**	150–10	5.50 ± 2.18	3.74	7.85	125 ± 49	76	175	0.64 ± 0.25	0.39	0.89
**IV.**	80	9.28	**II.**	110–10	4.77 ± 1.86	3.53	6.92	109 ± 42	72	149	0.56 ± 0.21	0.37	0.76
**V.**	60	6.96	**III.**	80–10	4.01 ± 1.49	3.19	5.74	92 ± 34	65	119	0.47 ± 0.17	0.33	0.61
**VI.**	40	4.64	**IV.**	60–10	3.37 ± 1.22	2.92	4.76	78 ± 29	60	103	0.40 ± 0.15	0.30	0.52
**VII.**	20	2.32	**V.**	40–10	2.60 ± 0.93	2.41	3.66	60 ± 21	49	76	0.31 ± 0.11	0.25	0.39
**VIII.**	10	1.16	**VI.**	20–10	1.69 ± 0.58	1.66	2.25	39 ± 13	34	47	0.20 ± 0.07	0.17	0.24

The mean values ± standard deviations and the ranges of CT dose index, dose length product, and effective dose are given for all scan protocol of the phantom and cadaver studies.

**Table 2:** Image quality (IQ) ratings, medians, and ranges of subjective image ratings of all scan protocols of the patient study as well as the ICC and *p* values of the comparisons are detailed in the table. Significant *p* values are indicated with *.

Protocols	Vertebra C3 Axial Sagittal										Vertebra C7 Axial Sagittal									
	Range Min Max		Median	*p* value	Cronbach’s α	Range Min Max		Median	*p* value	Cronbach’s α	Range Min Max		Median	*p* value	Cronbach’s α	Range Min Max		Median	*p* value	Cronbach’s α
**REFERENCE**	2	2	2	-	1.000	2	2	2	-	1.000	1	2	2	-	0.836	1	2	2	-	0.847
**STANDARD**	2	2	2	>0.99999	1.000	2	2	2	>0.99999	1.000	1	2	2	0.15700	0.729	1	2	2	0.52700	0.872
**I.**	1	2	2	0.15700	0.750	1	2	2	0.01600*	0.902	0	2	1	<0.00001 *	0.961	0	2	1	<0.00001 *	0.961
**II.**	1	2	2	0.25000	0.897	1	2	2	0.01600 *	0.943	0	2	1	<0.00001 *	0.884	0	2	1	<0.00001 *	0.923
**III.**	1	2	2	0.12500	0.919	1	2	2	0.00800 *	0.943	0	2	1	<0.00001 *	0.810	0	2	1	<0.00001 *	0.910
**IV.**	1	2	2	0.12500	0.919	1	2	2	0.00800 *	0.949	0	2	1	<0.00001*	0.865	0	2	1	<0.00001 *	0.919
**V.**	1	2	2	0.01600 *	0.789	1	2	2	<0.00001 *	0.938	0	2	0	<0.00001 *	0.789	0	1	0	<0.00001 *	0.832
**VI.**	1	2	2	0.00200 *	0.949	1	2	2	<0.00001 *	0.826	0	2	0	<0.00001 *	0.848	0	1	0	<0.00001 *	0.872

Medians and ranges of subjective ratings of all scan protocols of the patient study as well as ICC and *p* values of the comparisons are detailed in the table. Significant *p* values are indicated with *.

**Table 3:** Dose reduction for upper and lower c-spine and physical body parameters; the individual dose-reduced protocols allowing for diagnostic image quality for axial and sagittal reformations and the patients’ physical body parameters are detailed for each patient in this table.

Patients	Patients’ Physical Parameters							Protocols	
	BMI (kg/m^2^)	Height (m)	Weight (kg)	Lateral Diameter (cm)	Anteroposterior	Shoulder Position			
					Diameter (cm)	Anatomic	Nominal Value	Vertebra C3	Vertebra C7
						Level			
**1**	30.9	1.8	100	48	26	C4/5	3	**VI.**	**ST**
**2**	27.5	1.63	73	46	25	C4	3.5	**VI.**	**ST**
**3**	27.2	1.83	91	47	22	C4/5	3	**VI.**	**ST**
**4**	25.5	1.75	78	44	22	C5/6	2	**VI.**	**IV.**
**5**	24.9	1.69	68	46	19	“swimmer”		**VI.**	**V.**
**6**	23.8	1.8	71	45	20	C4/5	3	**VI.**	**II.**
**7**	21.9	1.92	80	44	17	C5/6	2	**VI.**	**V.**
**8**	21.7	1.83	73	44	16	C6/7	1	**VI.**	**V.**
**9**	21.4	1.87	75	46	20	C5/6	2	**VI.**	**V.**
**10**	20.3	1.66	56	40	18	C4/5	3	**VI.**	**IV.**

The individual dose-reduced protocols allowing for diagnostic image quality for axial and sagittal reformations and the patients’ physical body parameters are detailed for each patient in this table.

**Table 4:** High-contrast resolution in a corresponding phantom study; the table presents the high-contrast resolution of the phantom study; the values represent the gap size within two lines; the gap sizes were 0.045 mm, 0.042 mm, and 0.038 mm for a resolution of 11 lp/cm, 12 lp/cm, and 13 lp/cm, respectively.

Protocol no.	Tube current (mAs)	Reader—1	Reader—2	Reader—3	Reader—4	Reader—5
**STANDARD**	300	0.042	0.042	0.038	0.038	0.038
**I.**	150	0.042	0.042	0.038	0.038	0.038
**II.**	110	0.042	0.042	0.038	0.038	0.038
**III.**	80	0.042	0.042	0.042	0.042	0.038
**IV.**	60	0.042	0.042	0.042	0.042	0.042
**V.**	40	0.042	0.042	0.042	0.042	0.042
**VI.**	20	0.045	0.045	0.045	0.045	0.045
**VII.**	10	0.045	0.045	0.045	0.045	0.045

The table presents the high-contrast resolution of the phantom study; the values represent the gap size within two lines; the gap sizes were 0.045 mm, 0.042 mm, and 0.038 mm for a resolution of 11 lp/cm, 12 lp/cm, and 13 lp/cm, respectively.

## Data Availability

Data analysis was based on anonymized data from the picture archive system (PACS), radiology information system (RIS), hospital information system (HIS) and the general prosecutor’s data system.

## Abbreviations

body mass index	BMI
Canadian Cervical Spine Rule	CCR
cervical spine	c-spine
computed tomography	CT
computed tomography dose index	CTDI
dose length product	DLP
effective dose	ED
image quality	IQ
mean value	MV
National Emergency X-Radiography Utilization Study	NEXUS
standard deviation	SD
Statistical Package für Social Sciences	SPSS

## References

[1] Holly L.T., Kelly D.F., Counelis G.J., Blinman T., McArthur D.L., Cryer H.G. (2002). Cervical spine trauma associated with moderate and severe head injury: Incidence, risk factors, and injury characteristics. J. Neurosurg..

[2] Tian H.L., Guo Y., Hu J., Rong B.Y., Wang G., Gao W.W., Chen S.W., Chen H. (2009). Clinical characterization of comatose patients with cervical spine injury and traumatic brain injury. J. Trauma.

[3] Bayless P., Ray V.G. (1989). Incidence of cervical spine injuries in association with blunt head trauma. Am. J. Emerg. Med..

[4] McCaig L.F., Ly N. (2002). National Hospital Ambulatory Medical Care Survey: 2000 Emergency Department Summary—Advance Data from vital and Health Statistics.

[5] Davis J.W., Phreaner D.L., Hoyt D.B., Mackersie R.C. (1993). The etiology of missed cervical spine injuries. J. Trauma.

[6] Stiell I.G., Wells G.A., Vandemheen K.L., Clement C.M., Lesiuk H., De Maio V.J., Laupacis A., Schull M., McKnight R.D., Verbeek R. (2001). The Canadian C-spine rule for radiography in alert and stable trauma patients. JAMA.

[7] Stiell I.G., Clement C.M., McKnight R.D., Brison R., Schull M.J., Rowe B.H., Worthington J.R., Eisenhauer M.A., Cass D., Greenberg G. (2003). The Canadian C-spine rule versus the NEXUS low-risk criteria in patients with trauma. N. Engl. J. Med..

[8] ACR American College of Radiology Clinical Condition: Suspected Spine Trauma. http://www.acr.org/~/media/f579c123f999479c88390a3df976be77.pdf.

[9] Bailitz J., Starr F., Beecroft M., Bankoff J., Roberts R., Bokhari F., Joseph K., Wiley D., Dennis A., Gilkey S. (2009). CT should replace three-view radiographs as the initial screening test in patients at high, moderate, and low risk for blunt cervical spine injury: A prospective comparison. J. Trauma.

[10] Holmes J.F., Akkinepalli R. (2005). Computed tomography versus plain radiography to screen for cervical spine injury: A meta-analysis. J. Trauma.

[11] Hoffman J.R., Wolfson A.B., Todd K., Mower W.R. (1998). Selective cervical spine radiography in blunt trauma: Methodology of the National Emergency X-Radiography Utilization Study (NEXUS). Ann. Emerg. Med..

[12] Gale S.C., Gracias V.H., Reilly P.M., Schwab C.W. (2005). The inefficiency of plain radiography to evaluate the cervical spine after blunt trauma. J. Trauma.

[13] Mettler F.A., Thomadsen B.R., Bhargavan M., Gilley D.B., Gray J.E., Lipoti J.A., McCrohan J., Yoshizumi T.T., Mahesh M. (2008). Medical radiation exposure in the U.S. in 2006: Preliminary results. Health Phys..

[14] Larson D.B., Johnson L.W., Schnell B.M., Salisbury S.R., Forman H.P. (2011). National trends in CT use in the emergency department: 1995–2007. Radiology.

[15] Broder J., Warshauer D.M. (2006). Increasing utilization of computed tomography in the adult emergency department, 2000–2005. Emerg. Radiol..

[16] Richards P.J., George J., Metelko M., Brown M. (2010). Spine computed tomography doses and cancer induction. Spine.

[17] Geyer L.L., Körner M., Hempel R., Deak Z., Mueck F.G., Linsenmaier U., Reiser M.F., Wirth S. (2013). Evaluation of a dedicated MDCT protocol using iterative image reconstruction after cervical spine trauma. Clin. Radiol..

[18] Weinrich J.M., Regier M., Well L., Bannas P., Nykolyn O., Heinemann A., Sehner S., Behzadi C., Püschel K., Adam G. (2019). Feasibility of sub-milliSievert CT of the cervical spine: Initial results in fresh human cadavers. Eur. J. Radiol..

[19] Tozakidou M., Yang S.R., Kovacs B.K., Szucs-Farkas Z., Studler U., Schindera S., Hirschmann A. (2019). Dose-optimized computed tomography of the cervical spine in patients with shoulder pull-down: Is image quality comparable with a standard dose protocol in an emergency setting?. Eur. J. Radiol..

[20] Tozakidou M., Reisinger C., Harder D., Lieb J., Szucs-Farkas Z., Müller-Gerbl M., Studler U., Schindera S., Hirschmann A. (2018). Systematic Radiation Dose Reduction in Cervical Spine CT of Human Cadaveric Specimens: How Low Can We Go?. AJNR Am. J. Neuroradiol..

[21] Bongartz G., Golding S.J., Jurik A.G., Leonardi M., van Persijn van Meerten E., Rodríguez R., Schneider K., Calzado A., Geleijns J., Jessen K.A. 2004 CT Quality Criteria. European Guidelines for Multislice Computed Tomography. The European Commission. Contract number FIGM-CT2000-20078-CT-TIP..

[22] Wirth S., Meindl T., Treitl M., Pfeifer K.J., Reiser M. (2006). Comparison of different patient positioning strategies to minimize shoulder girdle artifacts in head and neck CT. Eur. Radiol..

[23] Wu T.H., Hung S.C., Sun J.Y., Lin C.J., Lin C.H., Chiu C.F., Liu M.J., Teng M.M., Guo W.Y., Chang C.Y. (2013). How far can the radiation dose be lowered in head CT with iterative reconstruction? Analysis of imaging quality and diagnostic accuracy. Eur. Radiol..

[24] Mueck F.G., Roesch S., Geyer L., Scherr M., Seidenbusch M., Stahl R., Deak Z., Wirth S. (2014). Emergency CT head and neck imaging: Effects of swimmer’s position on dose and image quality. Eur. Radiol..

[25] Kane A.G., Reilly K.C., Murphy T.F. (2004). Swimmer’s CT: Improved imaging of the lower neck and thoracic inlet. AJNR Am. J. Neuroradiol..

[26] Deak P.D., Smal Y., Kalender W.A. (2010). Multisection CT protocols: Sex- and age-specific conversion factors used to determine effective dose from dose-length product. Radiology.

[27] Niiniviita H., Kiljunen T., Huuskonen M., Teperi S., Kulmala J. (2018). Dose monitoring in pediatric and young adult head and cervical spine CT studies at two emergency duty departments. Emerg. Radiol..

[28] Holroyd J.R., Edyvean S. (2018). Doses from cervical spine computed tomography (CT) examinations in the UK. Br. J. Radiol..

[29] Vilar-Palop J., Vilar J., Hernández-Aguado I., González-Álvarez I., Lumbreras B. (2016). Updated effective doses in radiology. J. Radiol. Prot..

[30] Mettler F.A., Huda W., Yoshizumi T.T., Mahesh M. (2008). Effective doses in radiology and diagnostic nuclear medicine: A catalog. Radiology.

[31] Metaxas V.I., Messaris G.A., Lekatou A.N., Petsas T.G. (2019). Patient doses in common diagnostic X-ray examinations. Radiat. Prot. Dosim..

[32] Raine S., Twomey L. (1994). Posture of the head, shoulders and thoracic spine in comfortable erect standing. Aust. J. Physiother..

[33] Raine S., Twomey L.T. (1997). Head and shoulder posture variations in 160 asymptomatic women and men. Arch. Phys. Med. Rehabil..

[34] Liu R.R., Prado K.L., Cody D. (2008). Optimal acquisition parameter selection for CT simulators in radiation oncology. J. Appl. Clin. Med. Phys..

